# Image-derived mean velocity measurement for prediction of coronary flow reserve in a canonical stenosis phantom using magnetic particle imaging

**DOI:** 10.1371/journal.pone.0249697

**Published:** 2021-04-22

**Authors:** Robert Siepmann, Henning Nilius, Florian Mueller, Katrin Mueller, Claudio Luisi, Seyed Mohammadali Dadfar, Marcel Straub, Volkmar Schulz, Sebastian Daniel Reinartz

**Affiliations:** 1 Physics of Molecular Imaging Systems, RWTH Aachen University, Aachen, Germany; 2 Applied Medical Engineering, RWTH Aachen University, Aachen, Germany; 3 Experimental Molecular Imaging, RWTH Aachen University, Aachen, Germany; 4 Department of Diagnostic and Interventional Radiology, Uniklinikum Aachen, Aachen, Germany; Southeast University, CHINA

## Abstract

**Introduction:**

Aim of this study is to evaluate whether magnetic particle imaging (MPI) is capable of measuring velocities occurring in the coronary arteries and to compute coronary flow reserve (CFR) in a canonical phantom as a preliminary study.

**Methods:**

For basic velocity measurements, a circulation phantom was designed containing replaceable glass tubes with three varying inner diameters, matching coronary-vessel diameters. Standardised boluses of superparamagnetic-iron-oxide-nanoparticles were injected and visualised by MPI. Two image-based techniques were competitively applied to calibrate the respective glass tube and to compute the mean velocity: full-duration-at-half-maximum (FDHM) and tracer dilution (TD) method. For CFR-calculation, four necessary settings of the circulation model of a virtual vessel with an inner diameter of 4 mm were generated using differently sized glass tubes and a stenosis model. The respective velocities in stenotic glass tubes were computed without recalibration.

**Results:**

On velocity level, comparison showed a good agreement (*r*_FDHM_ = 0.869, *r*_TD_ = 0.796) between techniques, preferably better for 4 mm and 6 mm inner diameter glass tubes. On CFR level MPI-derived CFR-prediction performed considerably inferior with a relative error of 20–44%.

**Conclusions:**

MPI has the ability to reliably measure coronary blood velocities at rest as well as under hyperaemia and therefore may be suitable for CFR calculation. Calibration-associated accuracy of CFR-measurements has to be improved substantially in further studies.

## Introduction

Coronary artery disease (CAD) is accountable for the majority of deaths worldwide. According to estimates, CAD was the underlying cause of every 7th death in the US in 2018 [1], killing a total of 370000 people annually [2]. CAD is caused by atherosclerotic plaques in coronary arteries, most commonly due to prolonged high blood pressure, smoking, high blood fats and diabetes [3]. As a consequence, stenosis may evolve and limit blood supply of the left ventricle, especially under conditions of higher oxygen demands e.g. during exercise. If the stenosis is haemodynamically compromising or the plaque ruptures, death can occur due to arrhythmia or myocardial infarction [4]. As clinical symptoms appear mostly in advanced stage of the disease an early diagnosis is crucial. There are two important coronary flow parameters that are used to detect pathologic haemodynamics: fractional flow reserve (FFR) and coronary flow reserve (CFR). According to Hoef et al. [5], the former primarily detects predominant focal epicardial disease and the latter predominant microvascular disease, meaning that some coronary pathologies may exhibit discordances between FFR and CFR based on their pathophysiology, which occurs in up to 30–40% of cases [6]. The ongoing DEFINE-FLOW study [7] supports this assumption, as preliminary results showed that coronary lesions with a pathologic, pressure derived FFR but a normal CFR and vice versa are both associated with more adverse cardiac events than lesions with both normal FFR and CFR when treated medically. This emphasizes that both parameters should be considered to allow for a reliable, early diagnosis of CAD.

Invasive coronary angiography (ICA) currently represents the gold standard for classifying CAD, during which a pressure-derived surrogate of the fractional flow reserve (FFR_ICA_) is measured with a pressure-wire: the pressure ratio between aortic and the concerning post-stenotic coronary vessel under maximal hyperaemic conditions provided by injecting intra-arterial adenosine. A stenosis, labelled with FFR_ICA_ ≤ 0.75 has to be treated, mostly by stent implantation or bypass-surgery [8,9]. ICA comes with a regular radiation burden of 8 to 10 mSv per treatment [10] and potential massive radiation doses in complex recanalization procedures like chronic total occlusions [11] to which also the interventionalist is exposed to [10]. Furthermore, ICA relies on the use of iodinated contrast agents, leading to possible damage of the kidney [12] or allergic reactions [13,14]. Carbon dioxide or gadolinium may be used in special non-coronary cases, but they provide an inferior image quality and potential serious adverse effects [15,16]. Additionally, major complications like stroke, myocardial infarction, dissection or bleedings may occur due to invasiveness of the procedure. For these reasons, clinical admission to ICA should not be decided frivolously by physicians and a bouquet of non-invasive imaging modalities (i.e. drug-induced stress magnetic resonance imaging (cMRI) or echocardiography stress test (stress-ECHO), coronary computed tomography angiography (CCTA) or stress single-photon emission computed tomography (SPECT)) are recommended by guidelines [17] to detect coronary ischemia prior to invasive catheterisation. However, the examinations are time-consuming, expensive and cannot replace a consecutive ICA for definitive diagnosis. For these reasons, there are many approaches to avoid invasive measurements and to assess FFR and CFR by using non-invasive imaging [18].

Against this background, Magnetic particle imaging (MPI) could be an option, because this three-dimensional imaging modality addresses non-invasive functional imaging like CFR and has the prerequisite for interventional therapy [19]. Basically, MPI uses several different magnetic fields to detect the spatial distribution of superparamagnetic iron oxide nanoparticles (SPIONs). Firstly, the selection field is a static inhomogeneous magnetic gradient field, which saturates all SPIONs except those located in close proximity to the so-called field-free point (FFP), where no magnetic force is present. Secondly, the magnetic drive fields change sinusoidally in time, but are spatially homogeneous fields used to move the FFP across the region of interest and to excite the particles at the same time. Excited SPIONs induce a voltage in the receive coils and, eventually, the resulting current leads to a signal response. MPI comes with both high spatial and temporal resolution (i.e.21ms) [20] in the absence of ionizing radiation. By implementing modified scanner setups, even a submillimetre resolution is technically feasible [21,22]. However, to face the requirements of an upcoming technique, analysis of the blood velocity and flow should be used to characterize stenoses in the context of CAD as shown by Zafar et al. [23] using optical coherence tomography (OCT).

Hence, the aim of this study is to evaluate MPI’s ability to quantify velocities occurring in coronary vessels towards the aim of functionally characterizing the severity of a stenosis by the CFR. Thus, our approach is to generate realistic coronary flow rates in a closed water circuit and measure the resulting velocities with SPION-based MPI-image evaluation techniques. In a next step, as a preliminary study for CAD, a stenosis is assessed by CFR via flow-meter measurements of the circulation phantom as ground truth (CFR_GT_) and compared to CFR_MPI_ computed via MPI-image evaluation.

## Methods

### Theoretical considerations

**Fig 1:**
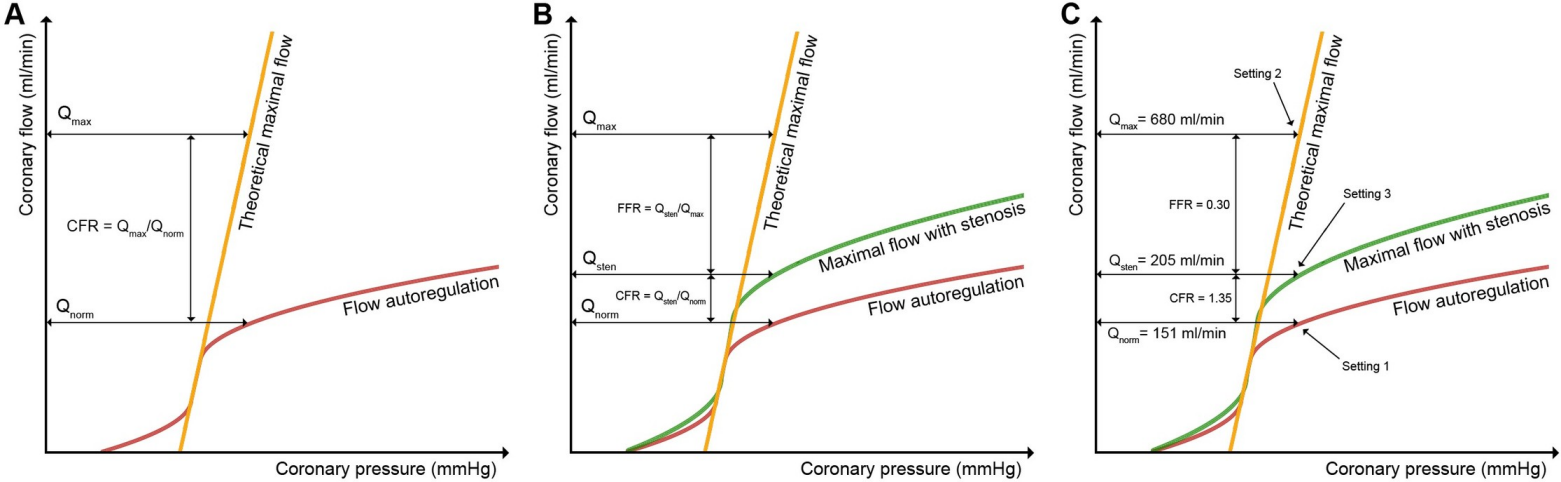
Schematic coronary flow/pressure diagrams. The diagrams visualize definitions of coronary flow reserve in the absence **(A)** or presence **(B)** of a haemodynamically relevant obstructing stenosis. In **(C)**, the experimental settings are denoted [7]. Setting 1 represents a healthy, non-dilatated vessel, in our case 4 mm inner diameter, e.g. right coronary artery, which is perfused with normal flow at rest (151 ml/min). Setting 2 represents an already dilated and hyperaemic perfused vessel (6 mm inner diameter) in the absence of a stenosis, simulating theoretical maximal flow of 680ml/min. Setting 3 represents the stenotic and therefore diseased vessel, perfused with hyperaemic flow and already dilated, resulting in a flow of 205 ml/min. In consequence, our simulated circulation phantom mimics a haemodynamic relevant stenosis with an FFR of only 0.30. In setting 4, the hyperaemic flow used in setting 3 is doubled to quantify very high flow in diseased vessels.

According to van de Hoef et al. [5,24] the coronary flow reserve (CFR) of a healthy vessel is defined by the ratio between theoretical maximal blood flow (Q_max:_: hyperaemic flow, dilated vessel) and blood flow at rest (Q_norm_: normal flow, non-dilated vessel). CFR is the factor by which the coronary blood flow can be increased if required (Fig 1A). Normal values are approximately 5 [25]. Under the condition of a flow-restriction due to a stenosis, CFR is impaired. The lost fraction of CFR is described by the fractional flow reserve (FFR or relative CFR) and defined by the ratio of maximal achievable flow under hyperaemia and dilatation in the presence of the stenosis (Q_sten_) and the theoretical maximal flow (Q_max_) as described earlier (Fig 1B). Values of the flow-derived FFR as well as the clinically used surrogate pressure-derived FFR_ICA_ are smaller than 1 and represent the severity of the stenosis. Values below 0.8 (main stem) or below 0.75 require revascularization in terms of clinical assessment [8,26].

In line with this concept and to finally simulate haemodynamically relevant stenoses, requirements to a coronary circulation phantom include coverage of coronary velocities between normal and hyperaemic conditions, a hemodynamically relevant degree of stenosis and a realistic extent of vessel dilation under hyperaemic conditions.

## Experimental setup

### Experiment A: Velocity evaluation in non-stenotic glass tubes

In the first step we build up a circulation model filled with water and designed it with coronary specific flow velocities ranging from 20 to 64 cm/s for the experiments. This covers the majority of the stenosis-relevant velocities present in the proximal part of the left anterior descending, left circumflex and right coronary artery in the resting state (31±15, 25±8, 26±7 cm/s, respectively) as well as during hyperaemia (66±18, 50±14, 48±13 cm/s, respectively) [27].

**Fig 2:**
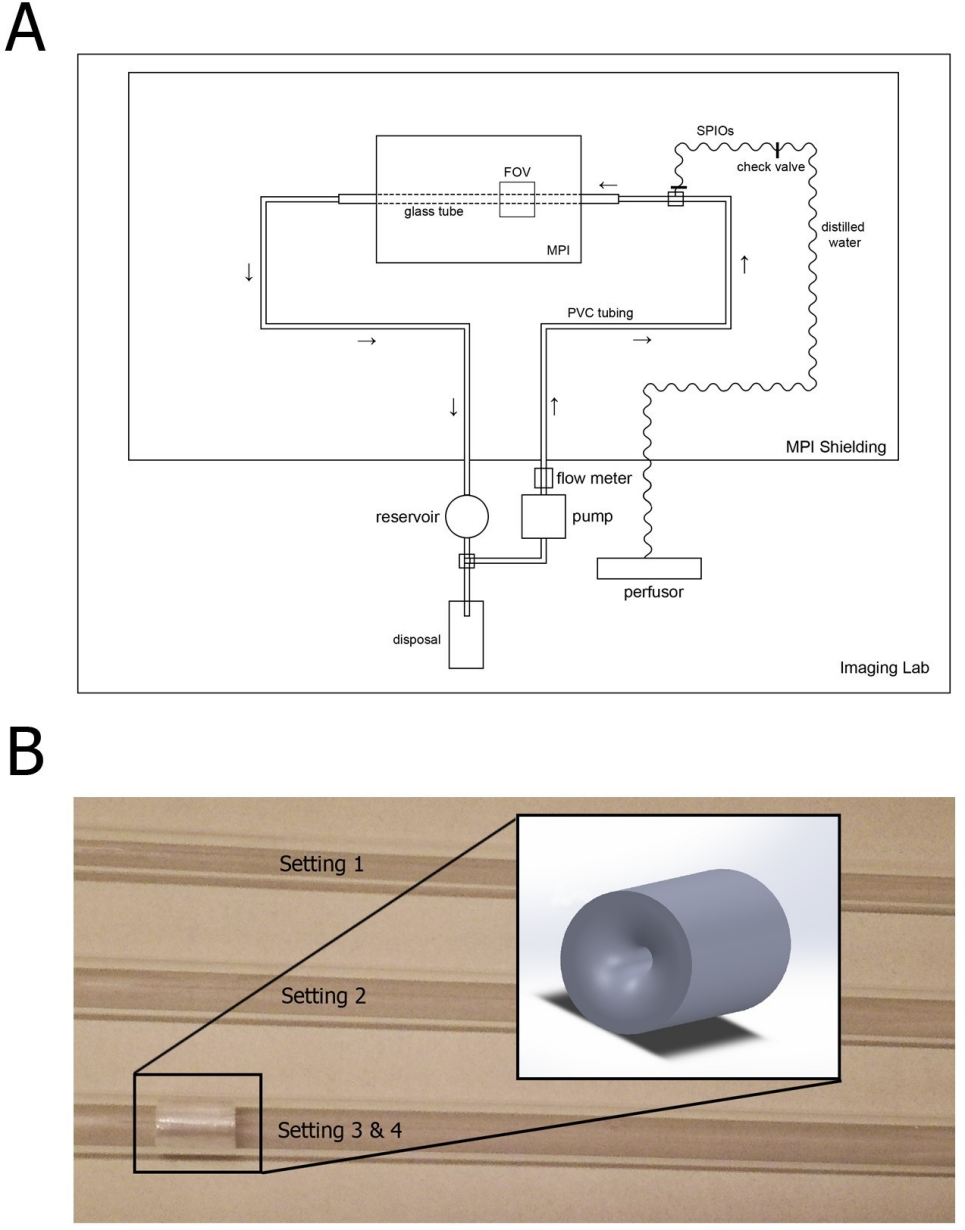
Experimental setup and stenosis model. **(A)** Glass tubes with an inner diameter of 4, 6 and 10 mm were used as imaging phantoms and connected to a centrifugal pump via dedicated tubes to establish a closed water circuit. An ultrasonic flow-meter was used to adjust the flow rates in order to obtain coronary-specific velocities inside the glass tubes. Standardised SPION-boluses were then injected using a perfusor and visualised by a preclinical MPI scanner. **(B)**: In experiment B four specific flow settings were investigated by using MPI to compute the coronary flow reserve in a simulated stenotic vessel (setting 1: 4 mm diameter, no stenosis; setting 2: 6 mm diameter, no stenosis; setting 3: 6 mm diameter, stenosis; setting 4: 6 mm diameter, stenosis, doubled flow rate as in 3).

The circulation phantom was inserted into a preclinical MPI scanner (MPI PreClinical 25/20FF, Bruker, Ettlingen, Germany [28,29], (Fig 2A) and filled with ultrapure water to avoid measurement artefacts due to contamination. It consists of a technical/electrical part outside of MPI’s Faraday cage to avoid interference and coupling with the MPI’s receive coils and the water-carrying tubular part. Despite of big plastic water pipes, the latter consisted of an exchangeable glass tube which ran parallel aligned to the x-axis through the MPI’s gantry to assure laminar flow. Three glass tubes with an inner diameter of 4 mm, 6 mm and 10 mm were examined, driven by a centrifugal pump (MEDOS Deltastream DP3, MEDOS Medizintechnik AG, Stolberg, Germany) to form a closed water circuit. Functionally, flow rates were adjusted by using an ultrasonic flow-meter (BioProTT™ FlowTrack plus, em-tec GmbH, Finning, Germany) attached outside to the afferent tube to obtain the above-mentioned velocity range inside the glass tubes as reference flows, defined as ground truth. The tube specific corresponding flow rates were calculated and ranged from 155 to 485 ml/min, from 340 to 1100 ml/min and from 962 to 3050 ml/min for glass tubes with an inner diameter of 4, 6 and 10 mm, respectively. Before each measurement, air bubbles were removed and the pump was left running for a few minutes to secure steady state water flow, validated by stable flow-meter values. SPIONs (perimag®, micromod, Berlin, Germany) with an iron content of 8.5 mg/ml were used as tracer material. Preliminary experiments with incremental bolus volumes at different flows showed that minimal tracer volumes of 0.3 ml were sufficient to generate a strong signal response in all used glass tubes even at high velocities. To safely provide sufficient image quality, 0.5 ml SPION-boluses were injected into the circulation phantom by a syringe pump (Perfusor fm, Braun®, Melsungen, Germany) in order to standardise tracer inflow. The distance between site of bolus injection and the field of view (FOV) amounted to approximately 25 cm due to scanner dimensions. The injected SPION boluses were then visualised continuously by the MPI scanner. After each bolus injection, the content inside the circulation phantom was replaced with new ultrapure water to avoid recirculation effects.

### Experiment B: CFR evaluation in stenotic glass tubes

#### Realization

In the second step we designed stenosis-specific settings with this circulation model to evaluate MPI’s capability to calculate CFR and FFR (or relative CFR). Experiment B was intended to be an addition to the flow measurements in experiment A while making as few changes in the experimental setup as possible to avoid additional MPI-related issues. The stenosis phantom was drilled out of plexiglass with a centered minimal lumen of 1.5 mm (length 10 mm, diameter 6 mm, Fig 2B) and fixed within the glass tube to avoid dislocation during increasing water pressure, demonstrated in a preliminary study. The stenosis was placed in the distal end of the glass tube to maximize the distance between the actual FOV and the stenosis. That way, turbulent flow conditions were limited to the area close to the stenosis whereas mainly laminar flow was assured in the FOV, comparable to experiment A.

The stenosis phantom was drilled out of plexiglass with a centered minimal lumen of 1.5 mm (length 10 mm, diameter 6 mm, Fig 2B) and fixed into the glass tube to avoid dislocation during increasing water pressure, demonstrated in a preliminary study.

Next, the behaviour of healthy and CAD-diseased virtual vessels was simulated. Therefore, we compared a ‘non-stenotic and non-dilated’ glass tube under normoaemic conditions (setting 1: 4 mm inner diameter) to a ‘non-stenotic’ tube (setting 2: 6 mm inner diameter), simulating hyperaemic flow in a healthy and dilated vessel. Finally, a 6 mm inner diameter tube with a stenosis was introduced, representing hyperaemic flow in a dilated and CAD-diseased vessel (setting 3). These three settings enable for calculation of CFR and FFR (Fig 1B and 1C) of a virtual vessel. MPI-based CFR calculation was validated by ultrasound-flowmeter measurements (ground truth).

To mimic the physiologic behaviour of the heart, the pump’s electrical power consumption needed for the establishment of hyperaemia in setting 2 was equally applied in setting 3, assuring that the resulting decrease in flow in the diseased vessel is attributed to the introduced stenosis. To simulate a haemodynamically less-relevant stenosis without affecting the delicate MPI setup, the electrical power of the pump was simply doubled (setting 4 = 2 x Setting 3 in hyperaemia). For each setting, at least three standardized SPION boluses (0.5 ml perimag) were injected and monitored via MPI as described in part A.

#### CFR-calculation

The calculation of CFR and FFR relies on the assumption that normaemic flow in non-stenotic tubes equals the one in stenotic tubes, as autoregulatory processes in the coronaries ensure a baseline flow in healthy as well as in diseased vessels to maintain proper oxygen supply. As a result, the baseline flow presented in setting 1 was referenced for CFR/FFR calculation in stenotic settings. CFR and FFR were calculated for image-derived measurements (CFR_MPI (set3&4)_, FFR_MPI(set3&4)_) and compared to the ground truth (flowmeter) (CFR_GT(set3&4)_, FFR_GT(set3&4)_) as defined in Fig 1B and 1C.

Velocities were calculated using the cross-correlated correction factor of the respective measurement series, representing the intrinsic correction factor (iCF). The velocities for setting 3 and 4 were calculated using iCF_set2_ to evaluate feasibility of non-stenotic tubes being used as a calibration for stenotic ones. Finally, the iCF_set3_ was used to calculate the velocities in setting 4 in order to assess calibration viability using stenotic tubes.

### Image acquisition

MPI datasets were acquired using 1 mm isotropic 3D settings with a drive field strength of (14,14,14) mT in (x,y,z)-direction and a gradient strength of 2.5 T/m. This led to a drive-field-FOV of 22.4×22.4×11.2 mm^3^ and to a System-matrix-FOV of 28×28×14 mm^3^. The temporal resolution of 21.54 ms in the context of dynamic measurements leads to image stacks with 46.43 images per second. For the x-channel a local receive coil was used in this study. Images were reconstructed by using the established Kaczmarz algorithm [30] with a regularisation factor of λ = 0.01 and without background correction. A dedicated system matrix was used, acquired in 16 hours with a probe volume of 1 μl by 134 averages (200 averages for local receive coil).

### Image evaluation

The reconstructed MPI images were analysed by a dedicated program written in python 3.6 (www.python.org).

#### Bolus identification

The dataset of a single bolus measurement is arranged in image sequences. Each one illustrates the signal intensity of a layer of the glass tube observed over time. This leads to (x, y, z, t) datasets, in which x, y, z represent the length in flow direction, the height and width, respectively, and t represents the time. A frame is defined as the entirety of all images at the same point of time.

At first, the signal intensity was summed up for every frame resulting in an intensity over time curve. The influence of an interpolation of the curve in terms of velocity calculation was tested in a controlled setup. Therefore, a virtual bolus shaped as a line (27 pixel × 1 pixel) with increasing intensity from both ends to the centre of the line was simulated. Hereby, the configuration of the simulated bolus matched the one in the reconstructed images as the dilution after injection leads to a distorted shape in longitudinal direction. Applying both algorithms on the simulated bolus showed superior results when using the interpolated curve, especially for higher velocities. A linear interpolation factor of 10 proved to be beneficial, which was then applied to the intensity over time curve. Instead of an advanced background subtraction [22], the baseline intensity was determined [31] and subtracted as a rudimental version of background correction. If the imaged bolus matched a typical bolus shape (no distortion, single intensity maximum), velocities of the bolus were calculated by using two different algorithms as described in the next section.

#### Bolus surveying techniques

**Fig 3:**
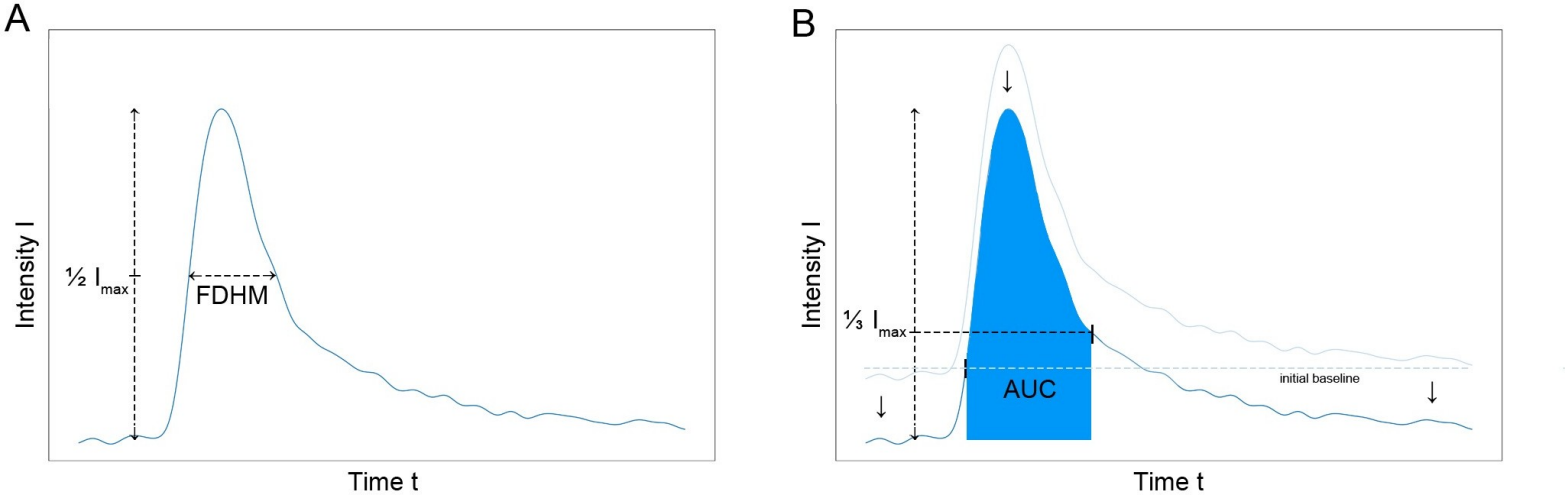
Algorithms for velocity calculation. **(A)** FDHM method: The FDHM is the time span at which at least half of the maximum intensity is present. The mean velocity can then be calculated by including the size of the FOV in longitudinal direction. **(B)** Tracer dilution method: The flow rate can be calculated by dividing a calibration constant by the area under the curve (AUC) of the signal intensity over time curve. The first border of the AUC is the intersection of the mean intensity of the initial baseline and the baseline subtracted intensity over time curve. The end of the AUC is defined as the frame at which 33% of the maximum signal intensity is present during signal decline after the peak. The computed flow rate can then by converted to the mean velocity by including the inner diameter of the phantoms.

The full duration at half maximum (FDHM) technique allows for quantification of different signal peaks. It is defined by the time span at which at least half of the maximum signal intensity is present (Fig 3A). The velocity *v* can be calculated by using a correction factor *c* and the size of the FOV in longitudinal direction *s* as shown in Eq (1).

$$v=\frac{c∙s}{FDHM}$$ (1)

The thermodilution method [32] (TD) is commonly used in intensive care medicine to monitor cardiac output with Swan-Ganz-catheters under usage of the Stewart-Hamilton’s equation [33]. The temperature of an intravenously administered agent is measured over time. By including the initial temperature of the blood *T_b_* and the tracer *T_i_*, the injected volume *V*, the area under the temperature-time-curve *AUC* and a specific correction factor *k*, the cardiac output can be calculated as shown in Eq (2).

$$Cardiac\;output=\frac{V∙(T_{b}-T_{i})∙k}{AUC}$$ (2)

Transferred to our experimental setup, we set the measured property from temperature to signal intensity. As the initial intensity of the FOV has already been subtracted, T_b_ equals 0. The injected tracer volume and its initial signal intensity as well as the constant *k* can be included in the new correction factor *ε*. The start of the *AUC* is defined as the frame at which the mean intensity of the initial baseline first crosses the baseline subtracted intensity over time curve (Fig 3B). The end of the *AUC* is defined as the frame at which 33% of the maximum signal intensity is present during signal decline after the peak. This is due to difficulty in determining the exact end of the peak because of background noise. Therefore, the flow rate *Q* of the circulation phantom can be calculated by using the following adapted formula (3):

$$Q=\frac{\varepsilon }{AUC}$$ (3)

As the cross section *A* of the respective glass tubes is known, the velocity can be calculated by using the formula of the volumetric flow rate as seen in Eq (4).

$$v=\frac{\varepsilon }{A∙AUC}$$ (4)

The iCFs *c* and *ε* are calculated as ratio to the experimental velocity (ground truth) for each measurement and cross-validated by the mean of the complementary correction factors of the concerning series. The error of the correction factor is the standard error of the mean of the remaining correction factors.

### Statistics

All statistical analyses were performed with IBM SPSS Statistics Version 24 (IBM Corporation, Armonk, NY). Categorical variables were expressed as counts and percentages, while continuous data was expressed as mean ± standard deviations. Pearson correlation coefficient was used to quantify the agreement with the reference values. Statistical significance was defined as a p-value <0.05.

## Results

### A: Velocity evaluation in the circulation model

**Fig 4:**
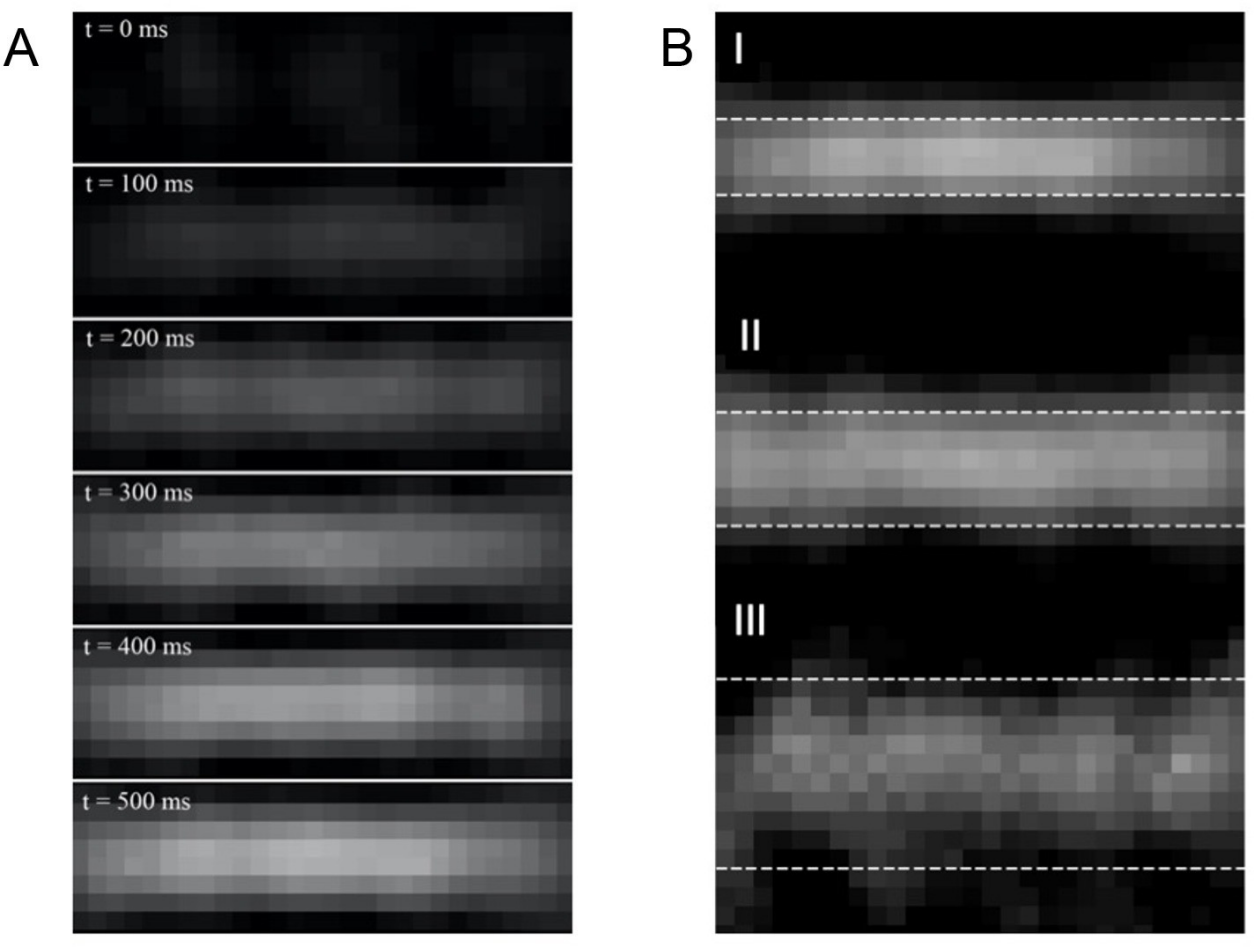
MPI-visualized bolus inflow. **(A):** MPI images of the bolus inflow in a glass tube with a 4 mm inner diameter in time steps of 100 ms. At first, only background noise is present. Subsequently, a line shaped central signal fades in, which saturates after a short period of time until a maximum is reached**. (B)**: Comparison of the bolus intensity peak between all three glass tubes at a velocity of 0.51 m/s. The dotted lines represent the presumable locations of the glass tube edges. The bolus of the (I) 4 mm inner diameter glass tube appears well-defined and more evenly distributed compared to the ones of the (II) 6 and (III) 10 mm inner diameter glass tube. With increasing diameter of the glass tube, the bolus becomes progressively inhomogeneous and the edges become more difficult to demarcate.

For each size of the glass tube, six 0.5 ml SPION-boluses were recorded by MPI to acquire image sequences of approximately 3000–9000 frames (64.6s -193.8s) each depending on the expected velocities. In each measurement, the bolus was identifiable in the reconstructed images and the peak intensity was determined. The calculated velocities using both algorithms in comparison to the ground truth (flow-meter) are shown in Table 1, an example of a bolus inflow is presented in Fig 4A. A comparison of the reconstructed MPI images between the three glass tubes at the signal intensity peak is shown in Fig 4B. For both algorithms, the deviation from the reference values rises with increasing inner diameter of the glass tubes, as expressed by absolute relative errors ranging from 0.7% to 23.3% (FDHM method) and from 2.5% to 45.6% (tracer dilution method) for the 4 mm inner diameter glass tube whereas the error margin of the 10 mm inner diameter glass tube covers values from 2.3% to 39.2% (FDHM method) and from 7.2% to 121.6% (tracer dilution method).

**Table 1:** MPI-derived velocities in non-stenotic phantoms.

Diameter [mm]	Flow rate [ml/min]	Reference velocity [cm/s]	FDHM [cm/s]	Correction factor *c* [1]	Absolute error [cm/s]/Absolute relative error FHDM	TD [cm/s]	Correction factor ε [ml]	Absolute error [cm/s]/Absolute relative error TD
4	155 ± 24.7	20.6 ± 3.8	25.4 ± 2.0	22.99 ± 1.24	4.8/23.3%	30.0 ± 1.8	31.44 ± 1.48	9.4/45.6%
	190 ± 25.7	25.2 ± 4.2	30.4 ± 2.6	23.58 ± 1.36	5.2/20.6%	20.8 ± 3.5	28.78 ± 2.06	4.4/17.5%
	230 ± 26.9	30.5 ± 4.6	24.1 ± 3.8	21.87 ± 0.84	6.4/21.0%	32.1 ± 3.9	30.04 ± 2.32	1.6/5.2%
	305 ± 29.2	40.5 ± 5.4	40.8 ± 4.8	22.91 ± 1.50	0.3/0.7%	39.5 ± 5.4	29.67 ± 2.33	1.0/2.5%
	385 ± 31.6	51.1 ± 6.4	50.5 ± 6.1	22.84 ± 1.49	0.6/1.2%	40.8 ± 7.2	28.59 ± 1.93	10.3/20.2%
	485 ± 34.6	64.3 ± 7.6	67.7 ± 5.9	23.07 ± 1.50	3.4/5.3%	70.5 ± 8.1	30.23 ± 2.29	6.2/9.6%
6	340 ± 30.2	20.0 ± 2.3	22.5 ± 1.3	18.46 ± 0.53	2.5/12.5%	35.3 ± 1.4	73.94 ± 3.47	15.3/76.5%
	430 ± 32.9	25.3 ± 2.6	25.5 ± 1.9	18.13 ± 0.65	0.2/0.8%	31.5 ± 2.9	70.84 ± 6.21	6.2/24.5%
	520 ± 35.6	30.7 ± 2.9	32.4 ± 2.2	18.28 ± 0.62	1.7/5.5%	28.3 ± 3.8	67.52 ± 6.44	2.4/7.8%
	690 ± 40.7	40.7 ± 3.6	36.6 ± 3.2	17.78 ± 0.53	4.1/10.1%	36.3 ± 5.1	67.10 ± 6.32	4.4/10.8%
	870 ± 46.1	51.3 ± 4.4	54.7 ± 3.7	18.31 ± 0.61	3.4/6.6%	44.6 ± 6.4	66.76 ± 6.20	6.7/13.1%
	1100 ± 53	64.8 ± 5.3	57.8 ± 5.1	17.76 ± 0.51	7.0/10.8%	56.3 ± 8.1	66.74 ± 6.19	8.5/13.1%
10	962 ± 48.9	20.4 ± 1.3	28.4 ± 2.9	13.07 ± 1.39	8.0/39.2%	45.2 ± 1.7	71.02 ± 4.19	24.8/121.6%
	1200 ± 56	25.5 ± 1.6	35.1 ± 3.6	13.05 ± 1.40	9.6/37.6%	20.1 ± 3.7	61.76 ± 7.67	5.4/21.2%
	1450 ± 63.5	30.8 ± 1.8	30.1 ± 4.8	12.40 ± 1.52	0.7/2.3%	23.5 ± 4.4	61.36 ± 7.48	7.3/23.7%
	1900 ± 77	40.3 ± 2.3	43.3 ± 6.2	12.59 ± 1.52	3.0/7.4%	43.2 ± 6.1	65.26 ± 8.21	2.9/7.2%
	2420 ± 92.6	51.4 ± 2.8	58.9 ± 7.8	12.73 ± 1.50	7.5/14.6%	57.8 ± 7.6	65.76 ± 8.14	6.4/12.5%
	3050 ± 111.5	64.7 ± 3.5	39.7 ± 5.2	11.23 ± 0.59	25.0/38.6%	52.2 ± 9.5	62.04 ± 7.79	12.5/19.3%

This table shows the velocities for the FDHM method and the tracer dilution (TD) method in comparison to the reference values. Velocity errors emerge from the error of the mean of the correction factors.

**Fig 5:**
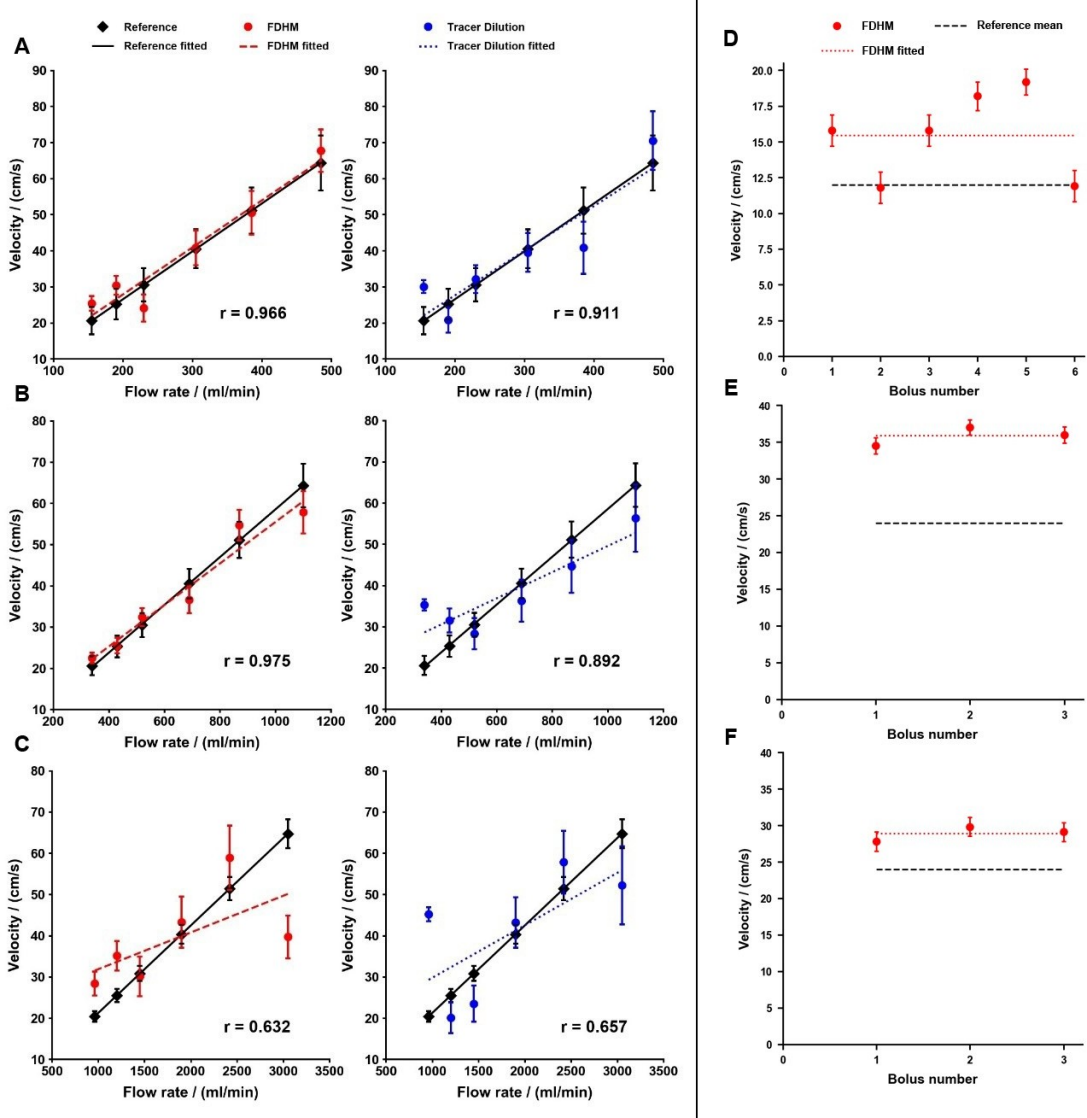
Computed velocities in comparison to the reference values of experiment A and B. Starting with experiment A, the first six plots show the velocities for the FDHM method and the tracer dilution method for glass tubes with an inner diameter of **(A)** 4 mm, **(B)** 6 mm and **(C)** 10 mm. The 4 mm diameter glass tube showed the best agreement for both algorithms, closely followed by the 6 mm diameter glass tube. The results were considerably inferior for the 10 mm diameter glass tube. The last three plots represent experiment B: Applying the correction factor of setting 2 on the measurements of setting 3 **(D)** and setting 4 **(E)** yields a mean relative error of 28% and 48% compared to the reference values, respectively. The lowest mean relative error of 20% is achieved when using the correction factor of setting 3 for setting 4 **(F)**. Error bars emerge from the error of the mean of the correction factors.

The results of the FDHM method of the 6 mm inner diameter glass tube shows the lowest relative errors of all measurement series (0.8% to 12.5%). For all sizes of glass tubes, the absolute relative error ranges of the tracer dilution method are markedly higher compared to FDHM method. The computed velocities and their respective linear curve fits for the FDHM method and the tracer dilution method in comparison to the reference values are shown in Fig 5. For both algorithms the Pearson’s correlation factor decreased with increasing inner diameter (Table 2). The correlation factors of the 4 mm diameter glass tube amount to r_FDHM_ = 0.966 and r_dilution_ = 0.911 (p < 0.05). Measurements in the 6 mm diameter glass tube lead to correction factors of r_FDHM_ = 0.975 and r_dilution_ = 0.892 (p < 0.05). The correlation factors of the 10 mm diameter glass tube are r_FDHM_ = 0.632 and r_dilution_ = 0.657 (p > 0.05). In total, the average Pearson correlation coefficient of the FDHM method (r_FDHM_ = 0.869, p < 0.05) for all size of glass tubes is slightly superior to the one of the tracer dilution method (r_dilution_ = 0.796, p < 0.05).

**Table 2:** Correlation factors of MPI-derived velocities.

Diameter [mm]	r_FDHM_	p_FDHM_	r_dilution_	p_dilution_
4	0.966	0.002	0.911	0.012
6	0.975	0.001	0.892	0.017
10	0.632	0.178	0.657	0.156

This table shows the Pearson’s correlation factors of the FDHM and TD method for the three different glass tubes. For the 4 mm and 6 mm inner diameter glass tubes, MPI-derived velocities showed good agreement with the reference ones. In contrast, computed velocities yielded inferior results for the 10 mm inner diameter glass tube.

### B: CFR-evaluation

By our experimental setting, we generated a CFR_GT_ = 4.50 as defined by setting 1 and 2 (Fig 1C) with respective flows of 151 ml/min and 680 ml/min. By introducing the stenosis into the glass tube in setting 3, we observed a mean flow of Q_GT-Set3_ = 205 ml/min, which generates a FFR_GT_ = 0.30 and a CFR_GT_ of 1.36. By doubling the flow in setting 4, the stenosis virtually diminishes, resulting in an FFR_GT_ = 0.60 and a CFR_GT_ = 2.72.

An overview of the results of the respective settings can be found in Table 3 and Fig 5D–5F.

**Table 3:** MPI-calculated velocities in stenotic phantoms.

Setting	Flow rate [ml/min]	Reference velocity [cm/s]	FDHM velcoties using intrinsic correction factor [cm/s]	Correction factor *c* [1]	Absolute error [cm/s]/Absolute relative error	FDHM velocities using correction factor from setting 2	Absolute error [cm/s]/Absolute relative error	FDHM using correction factor from setting 3	Absolute error [cm/s]/Absolute relative error
1	151 ± 24.5	20 ± 2.2	19.8 ± 2.0	25.30 ± 0.19	0.2/1.2%				
			21.5 ± 1.8	25.84 ± 0.08	1.5/7.7%				
			19.0 ± 2.0	25.03 ± 0.14	1.0/5.1%				
			19.8 ± 2.0	25.32 ± 0.19	0.2/0.8%				
2	680 ± 40.4	40 ± 3.6	42.7 ± 2.8	21.22 ± 0.13	2.7/6.8%				
			42.3 ± 2.9	21.18 ± 0.14	2.3/5.7%				
			34.9 ± 3.1	20.49 ± 0.09	5.1/12.9%				
			42.3 ± 2.9	21.18 ± 0.14	2.3/5.7%				
			42.4 ± 2.9	21.19 ± 0.14	2.4/6.0%				
			36.8 ± 3.1	20.69 ± 0.13	3.2/8.0%				
3	205 ± 26.2	12 ± 1.7	12.9 ± 1.6	17.09 ± 0.32	0.9/7.1%	15.8 ± 1.1	3.8/31.6%		
			9.0 ± 1.5	16.00 ± 0.25	3.0/25.2%	11.8 ± 1.1	0.2/1.9%		
			12.9 ± 1.6	17.10 ± 0.32	0.9/7.5%	15.8 ± 1.1	3.8/32.0%		
			15.2 ± 1.4	17.51 ± 0.29	3.2/26.6%	18.2 ± 1.0	6.2/51.8%		
			16.1 ± 1.3	17.65 ± 0.27	4.1/34.4%	19.2 ± 0.9	7.2/59.9%		
			9.1 ± 1.5	16.04 ± 0.26	2.9/24.2%	11.9 ± 1.1	0.1/0.9%		
4	410 ± 32.3	24 ± 2.5	22.7 ± 1.6	13.79 ± 0.06	1.3/5.6%	34.5 ± 1.1	10.5/43.7%	27.8 ± 1.3	3.8/15.7%
			25.2 ± 1.6	14.28 ± 0.11	1.2/4.8%	37.0 ± 1.0	13.0/54.1%	29.8 ± 1.3	5.8/24.0%
			24.3 ± 1.7	14.11 ± 0.17	0.3/1.1%	36.1 ± 1.1	12.1/50.4%	29.1 ± 1.3	5.1/21.1%

This table shows the velocities for the FDHM method in comparison to the reference values for the four prior specified settings (1: 4 mm diameter, no stenosis; 2: 6 mm diameter, no stenosis; 3: 6 mm diameter, stenosis; 4: 6 mm diameter, stenosis, doubled flow rate as in 3). At first, FDHM velocities were calculated using the cross-correlated correction factor of the respective measurement series, representing the iCF. In a next step, FDHM velocities for setting 3 and 4 were calculated by using the correction factor of setting 2 to evaluate feasibility of non-stenotic tubes being used as a calibration for stenotic ones. Finally, the correction factor of setting 3 was used to calculate the FDHM velocities in setting 4 in order to assess calibration viability using stenotic tubes. TD-method was not used due to inferior performance in comparison to FDHM.

The use of iCFs lead to FDHM-calculated velocities with good agreement to the reference ones, with a mean relative error of 6.9% overall and cleaned for outliers. Applying the mean correction factor of setting 2 for velocity calculation in a stenotic tube with hyperaemia (setting 3) and double hyperaemia (setting 4), the mean relative error amounts to 43.9%. Analogously, copying the mean correction factor of setting 3 to setting 4, the mean relative error yields 20.3%.

On the CFR-level, using the analogous mean correction factor of setting 2 for measurement of FFR and CFR of stenosis in setting 3 leads to FFR_set3_(c_set2_) = 0.39 (Δ_abs_ = 0.09; Δ_rel_ = 28%) and CFR _set3_(c_set2_) = 1.74 (Δ_abs_ = 0.38; Δ_rel_ = 28%) respectively. For stenosis evaluation in setting 4, applying the same correction factor delivers FFR _set4_(c_set2_) = 0.89 (Δ_abs_ = 0.29; Δ_rel_ = 48%) and CFR_set4_(c_set2_) = 4.02 (Δ_abs_ = 1.32; Δ_rel_ = 48%). Applying the mean correction factor of setting 3 for the measurements in setting 4 provides FFR_set4_(c_set3_) = 0.72 (Δ_abs_ = 0.12; Δ_rel_ = 20%) and a CFR _set4_(c_set3_) = 3.25 (Δ_abs_ = 0.53; Δ_rel_ = 20%).

## Discussion

This study was designed to systematically evaluate velocity quantification in a canonical coronary stenosis phantom with MPI for enabling measurements of coronary- or fractional flow reserve (CFR/FFR). In the first step, the measurement of incremental flow velocities in differently sized glass tubes via MPI as a surrogate for healthy vessels was performed to robustly allow quantification of mean velocities. We chose straight tubes to provide laminar flow which can be described mathematically by analytic models for validation purposes. Therefore, we avoided more complex phantoms like aneurysms or stenoses. Two robust techniques (FDHM, TD) successfully established in medical applications, were applied to evaluate the bolus velocities, which worked well and showed good agreement for 4 mm and 6 mm diameter glass tubes. We therefore believe to have successfully demonstrated the feasibility of calculating coronary-specific velocities as a proof of concept in MPI. Against this background, we hypothesized that MPI is potentially capable of identifying pathologic haemodynamics in the coronaries, especially CFR. Therefore, we simulated a diseased virtual coronary vessel with a CFR_GT_ = 4.50 by introducing a centered stenosis phantom into the glass tube. By defining four vessel specific settings in the glass tubes (Fig 1), the flow-states of the virtual coronary stenosis phantom were made accessible for MPI and were validated independently by the flow-meter measurements as our gold standard.

Our results show that the technique works sufficiently accurate to identify highly haemodynamically relevant stenosis as shown by a MPI-derived CFR value of 1.74 in setting 3. Due to the upgradable precision in CFR calculation, this method is currently unable to distinguish between adjacent conditions requiring treatment or not as demanded by clinical FFR thresholds.

This concept currently seems to work when the calibration is performed on the same level of complexity as the subsequent measurement: meaning non-stenotic calibrations will only work sufficiently in non-stenotic tubes and vice versa. Therefore, not only the diameter of the vessel but also the introduction of a stenosis, which eventually leads to a hydrodynamic change, seem to have a significant, still not fully understood influence on the iCF. Therefore, we assume that a thorough assessment of the iCF could be the key for a reliable CFR prediction using MPI. Since up to 60% of participants of FAME II study, eligible for percutaneous coronary intervention (PCI) with pathologic FFR, did not need revascularization in the follow-up period of 2 years [34], our accuracy seems to be similar to pressure-wire guided FFR predictions of ischemia. On the other hand, approximately 10% of FAME II patients suffered major adverse cardiac events although stratified by normal FFR–representing false negatives [34].

So far, other studies focused on flow quantification of aneurysm phantoms [28,35] or flows appearing in the venous vasculature. In a recent study by Kaul et al. [36] the mean blood velocity in the inferior vena cava of mice was calculated. Investigated velocities extend to 21 cm/s, which is the lower border of velocities in coronary vessels and therefore provides only partial information about MPI’s feasibility of coronary imaging. Thus, our study represents a convenient extension in velocity tracking using MPI by focusing on high velocity flow measurements suitable for cardiac or coronary imaging. Other groups have used MPI to image perfusion in *in vivo* experiments [37,38]. Alternatively, in another concept, the combination of measuring both flow and pressure in the concerning vessel allows for calculating the basal stenosis resistance index (BSR) [39] to characterize the severity of a stenosis functionally. This is feasible via this MPI study as well by using modified Bernoulli’s Eq (5) [40], the velocity can be translated into a pressure gradient, too.

Δp=4∙v² (5)

However, there are many limitations in our study: Firstly, the correlation for 10 mm diameter glass tube was surprisingly inferior, which may be caused by the increased inhomogeneity during bolus inflow in tubes violating laminar flow conditions with large inner diameter. Increasing the characteristic length, in this case the tube diameter, and increasing the flow rate will both lead to higher Reynolds numbers. As a result, turbulent flow may most likely be present in the 10 mm glass tube at high velocities. Taking into consideration the 25 cm long distance between injection and FOV and the constant bolus volume for all three sizes of glass tubes, this turbulence results in a significant tracer dispersion, which violates our assumption for laminar flow. This is reflected in Fig 4B, which shows that the boluses of the 4 mm and 6 mm diameter glass tubes are considerably more well-defined than the one of the 10 mm diameter glass tube. Taking into account that the average diameters of coronary arteries in healthy patients and in patients with CAD are 3.10 mm and 2.79 mm, respectively [41], the inferior results of the 10 mm diameter tube will probably not be a restriction for cardiovascular imaging using MPI.

Due to our simplified study design to develop a reliable tool for intracoronary velocity measurements, there are many general limitations. The straight glass tube used in the experiments represents the maximal simplification of coronary phantoms as it overlooks factors like branching, tortuosity and elasticity of vessel walls as well as pulsatile flow. The introduction of branching and tortuosity will have a considerable impact on the flow conditions inside the phantom, making a computation of the real flow velocity for validation purposes difficult. We therefore used straight and non-branching tubes to allow for a precise computation of reference velocities by using basic fluid mechanics. Furthermore, coronary walls are elastic in nature and not rigid like glass tubes as long as there is no advanced atherosclerotic disease, which would dynamically lower the pressure inside the phantoms leading to different flow conditions. Again, a reliable computation of the reference velocity for calibration purposes would be problematic as well. When introducing pulsatile flow, the SPIONs would fill up the phantom stepwise, but the intensity over time curve will not change its basic configuration, it will rather transform into a superimposed staircase figure of the curve using non-pulsatile flow. As the area under the curve and the full duration at half maximum do not change fundamentally, the algorithms would presumably work under these conditions as well. Since this is a pilot study to evaluate general feasibility of MPI for velocity computation, we chose to design the phantom as simple as possible in order to avoid additional sources of error that would arise when using realistic coronary phantoms. After a thorough assessment of MPI-derived flow characterization and its limitations on the most basic level, more realistic phantoms should be investigated in further studies.

Regarding MPI-derived CFR-evaluation, measuring velocities at places in which laminar flow is present is a limitation, too, as real atherosclerotic vessels do not have a long, straight and regular start-up length allowing for laminar conditions at the FOV. Realistically, it would be ideal to place the FOV insight the stenosis to allow for a reliable quantification as long as the imaging modality offers a sufficiently high enough resolution. We decided against this approach, as we wanted to change as few factors as possible compared to the basic flow measurements in experiment A and due to the fact that our MPI-scanner does not provide a mechanism to reliably and reproducibly assign the FOV to the desired location accurate to a millimeter as it would be necessary for our stenotic model.

Additionally, the use of water instead of blood is a limitation as well. As a consequence, we were not able to measure flow rates below 20 cm/s because the SPIONs start to sediment at low flow rates and therefore do not reflect the actual water flow anymore. This is a phenomenon that should be addressed when using real blood rather than water as this will most likely have a considerable influence on SPION sedimentation.

In sum, this study tries not to simulate a real-world scenario in the clinics like intravenous injection or catheter associated intracoronary injections, but to prove the validity of coronary relevant velocity measurements. Finally, a clinical application of the proposed technique might lead to sensitivity issues of MPI, and other system concepts [42–44] of MPI as proposed might be used.

## Conclusion

MPI is supposed to be a promising imaging modality that has the potential to comprehensively assess and treat CAD. As a first step, this preliminary study presented a successful quantification of coronary-specific velocities in simple geometries and an approach for calculation of CFR in canonical stenosis settings as a rough proof of concept. To allow for a reliable MPI-derived CAD assessment, calibration-associated accuracy of CFR-measurements has to be improved substantially in further studies.
